# Serum biochemical parameters as a surrogate marker for chest computed tomography in children with COVID-19

**DOI:** 10.2217/fvl-2021-0118

**Published:** 2021-09-02

**Authors:** Karuna M Das, Rajvir Singh, Sandeep Subramanya, Shreesh Kumar Ojha, Taleb Almansoori, Dilip Gokhale, Jamal Aldeen Alkoteesh

**Affiliations:** 1 Department of Radiology, CMHS, UAEU, Al Ain, UAE; 2 Department of Biostatistics, AIIMS, New Delhi, India; 3 Department of Physiology, CMHS, UAEU, Al Ain, UAE; 4 Department of Pharmacology, CMHS, UAEU, Al Ain, UAE; 5 Department of Radiology, Tawam Hospital, Al Ain, UAE

**Keywords:** COVID-19, Chest CT, LDH, serum ferritin

## Abstract

**Aim:**

This study aimed to investigate whether serum biochemical parameters can be used as a surrogate for chest computed tomography (CT) in the diagnosis of COVID-19 in pediatric patients.

**Materials & methods:**

We evaluated potential associations between various serum biochemical markers and the COVID-reporting and data system (RADS) pneumonia grading system in 53 individuals with confirmed COVID-19.

**Results:**

A total of 28 chest CT scans (52.8%) were abnormal. Patients with confirmed COVID-19 on CT showed a statistically significant increase in lactate dehydrogenase (186.4 ± 56.5 vs 228.4 ± 60.6; p = 0.01), which was significantly correlated with the COVID-RADS pneumonia grading system.

**Conclusion:**

Lactate dehydrogenase can be used as a surrogate marker for chest CT in children with COVID-19. This can reduce exposure to ionizing radiation during initial diagnostic procedures in children with suspected COVID-19 pneumonia.

COVID-19 pneumonia in children is associated with considerable morbidity and mortality [1,2]. Radiological examinations, particularly chest radiography and computed tomography (CT), have significantly aided in clinical diagnosis, evaluation and management of COVID-19 patients. A quantitative RT-PCR test is the gold standard for initial diagnosis, and it is frequently supplemented by chest radiography and CT for the evaluation of COVID-19 pneumonia [3–6].

Unfortunately, these imaging tests are associated with ionizing radiation exposure to this vulnerable group of patients [7–9]. Therefore, it is necessary to explore alternative convenient, safe and cost-effective diagnostic tests that could be used as surrogates for ionizing radiation modality, such as chest CT. Consequently, these alternative tests may help differentiate patients’ characteristics and thus prioritize clinical care for pediatric patients of COVID-19 based on serum biochemical parameters.

COVID-19 infection in children and adult populations is evolving, and it has been predicted that vaccinations are bound to help adults and geriatric populations to recover fast following infection. However, newer COVID-19 variants are expected to impact the unvaccinated pediatric population. Consequently, due to the fact that the pediatric population can be significantly more vulnerable to COVID-19, researchers were encouraged to explore the availability of safe, convenient, feasible and cost-effective diagnostic biomarkers for early disease assessment. Since the emergence of the pandemic, several reports have presented diagnostic clues on the altered serum biochemical parameters following the pathophysiological and biochemical changes in the pathogenesis of COVID-19 [10–12]. To date, no scientific study has managed to provide solid evidence on the relationship between chest CT findings and serum biochemical parameters in pediatric patients with COVID-19 pneumonia.

Therefore, this study aims to investigate the relationship between chest CT findings and serum biochemical parameters in vulnerable pediatric COVID-19 patients to determine whether these parameters can aid in the detection of early biochemical changes in the patients’ body. Positive correlation between changes in serum biochemical parameters and disease status can, subsequently, reduce exposure to ionizing radiation, which is a prerequisite in conventional chest CT imaging.

## Materials & methods

### Patient population

All data were retrospectively gathered between 16 March 2020 and 1 June 2020. The following criteria were used to determine inclusion: a positive RT-PCR test for SARS-CoV-2; at least one CT chest examination; and serum biochemical parameters. Exclusion criteria included individuals with flu-like symptoms and a negative RT-PCR test for SARS-CoV-2, as well as individuals without an initial chest CT. On the first day of hospitalization, pediatric patients had a chest CT and a complete blood count. The included serum biochemical values were determined according to the instructions of the treating physician on the day of admission. Following a thorough examination of medical files, clinical data (comorbidities, chest radiographic assessments and laboratory results) were retrieved from electronic medical records. Laboratory assessments comprised aspartate aminotransferase, alanine aminotransferase (ALT), lactate dehydrogenase (LDH), C-reactive protein, ferritin, white cell count, serum creatinine and lymphocyte count. All medical records were carefully reviewed, and data were abstracted before being entered into a computerized database and cross checked. Patients’ medical records were further reviewed with respect to their length of hospital stay and outcomes.

### Imaging technique

All chest CT scans were performed with a 64-slice helical CT scanner (Siemens Healthcare’s Sensation 64, Munich, Germany) using a tube kilovoltage (kV), 100–120 kV; tube current (mAs), automatic exposure management; collimation, 2.0 mm; pitch, 1; reconstruction algorithm, iterative-based reconstruction; reconstruction slice thickness, 0.5 mm; and interslice gap, 0 mm and reformatted with lung (width, 1500 HU; level, -500 HU) and soft tissue (width, 350 HU; level, 50 HU) window settings. CT images were acquired in a single breath hold at end inspiration with the subject in the supine position. Lungs were scanned from the thoracic inlet to the level of the diaphragm [5].

### Imaging study evaluation & categorization

Two radiologists with over 20 years of experience (Karuna M Das, Jamal A Al Kottesh) independently reviewed chest CT images in a picture archiving and communication system (Cedara I-Read 5.2 P11, Cerner Imaging Devices, MO, USA) in a random order. All conflicting reports were independently assessed by an arbitrator (Taleb Al Mansoori). Diagnosis of COVID-19 pneumonia was made in accordance with the criteria of CT findings of COVID-19 pneumonia in children from the international expert consensus statement on chest imaging in pediatric COVID-19 patient management [3,4,6]. Reviewers then assessed chest CT images for abnormalities in the thoracic structures, including: lung parenchyma; airway; pleura; and mediastinum, based on previously established criteria [3,4,6,13]. Patients with positive RT-PCR for COVID-19, ground-glass opacity, consolidation, nodular opacity, reticular markings, halo sign, reverse halo sign, crazy-paving pattern and the tree-in-bud pattern were noted as patients with COVID-19 pneumonia [13,14]. These patients were then classified into two subgroups (group 1: without pneumonia and; group 2: pneumonia). The CT features for COVID-19 pneumonia were graded as per the COVID-RADS data system from 1 to 5, as described earlier [15]. Correlations between the level of the serum biochemical parameters and the COVID-RADS grading system among the patients were evaluated.

### Statistical methods

Descriptive statistics were performed with mean with standard deviations and median with interquartile range for interval variables, and frequency distribution with percentages was calculated for categorical variables. Unpaired Student’s *t*-tests were used to compare the two groups for normally distributed interval variables and the Mann–Whitney U test for non-normal distributed variables. Interobserver reliability was calculated by Cohen’s kappa statistics (k) to assess the degree of agreement between the two reviewers. To study the association between LDH and COVID-RADS CT scores, the Eta coefficient test was performed. A p-value ≤ 0.05 (two-tailed) was considered statistically significant. SPSS 22.0 statistical software was used for the analysis.

## Results

### Patient cohort

The final sample population included 53 chest CT examinations obtained from 53 pediatric patients ranging in age from 7 months to 18 years (mean standard deviations: 13.21 ± 5.7 years). Group 1 included 25 of 53 (47.1%) pediatric patients (mean age: 11.81 ± 6.4 years; age range: 7 months–18 years; M:F 16:9) with no clinical evidence of pneumonia on chest CT. Group 2 included 28 of 53 (52.8%) pediatric patients (mean age: 14.46 ± 4.9 years; age range: 9 months–18 years; M:F 16:12) with confirmed pneumonia on chest CT (Table 1). There was no noticeable difference in the gender or age between the two groups (Table 2). The mean length of patients’ symptoms before hospitalization was 3.3 ± 1.2, with a range of 1–6 days. All patients were held in a quarantine facility for 15 days after hospital release, including the days spent in the hospital settings.

**Table 1.** Chest computed tomography findings in 53 symptomatic pediatric patients with COVID-19.

Chest CT findings	Studies, n (%)
Normal chest CT	25 (47.1%)
Abnormal chest CT	28 (52.8%)
Type of abnormal chest CT findings	
Peribronchial thickenings	0 (0%)
GGO	6 (21.4%)
Consolidation	2 (7.1%)
Peribronchial thickenings and GGO	0 (0%)
Peribronchial thickenings and consolidation	0 (0%)
GGO and consolidation	20 (71.4%)
Peribronchial thickenings, GGO and consolidation	0 (0%)
Signs and patterns of chest CT findings	
Halo sign	10 (35.7%)
Reversed halo sign	7 (25%)
Crazy-paving pattern	2 (7.1%)
Tree-in-bud pattern	1 (3.5%)
Air bronchogram	1 (3.5%)

CT: Computed tomography; GGO: Ground-glass opacity.

**Table 2.** Comparison of serum biochemical parameters as per computed tomography results in 53 patients.

Variable	Chest CT	n	Mean	p-value
Platelet count	Group 1	25	292.5 ± 76.8	0.04
150–350 × 109/l	Group 2	26	250.6 ± 68.7	
LDH	Group 1	25	186.4 ± 56.5	0.01
60–100 U/l	Group 2	26	228.4 ± 60.6	
AST	Group 1	23	23.6 ± 9.0	0.23
0–35 U/l	Group 2	24	29.2 ± 19.9	
ALT	Group 1	23	21.2 ± 12.2	0.99
0–35 U/l	Group 2	24	21.1 ± 8.5	
White count cell	Group 1	23	21.2 ± 12.2	0.99
4–10 × 10^9^ cells per l	Group 2	24	21.2 ± 8.5	
C protein	Group 1	25	4.4 ± 8.5	0.78
0.3–1.0 mg/dl	Group 2	26	5.1 ± 9.0	
Lymphocyte count	Group 1	25	3.3 ± 2.9	0.38
0.8–5.0/mcl	Group 2	26	5.1 ± 9.0	
Ferritin	Group 1	25	62.5 ± 37.4	0.08
15–200 mg/l	Group 2	25	119.6 ± 154.4	
Age (years)	Group 1	25	11.81 ± 3.6	0.1
	Group 2	28	14.46 ± 4.90	
Gender^†^	Group 1	25	M: 19, F: 6	0.25
	Group 2	28	M: 16, F: 12	

† Gender is reported as the number of males and females in each group.

ALT: Alanine aminotransferase; AST: Aspartate aminotransferase; CT: Computed tomography; LDH: Lactate dehydrogenase.

### Chest CT findings

**Figure 1.**
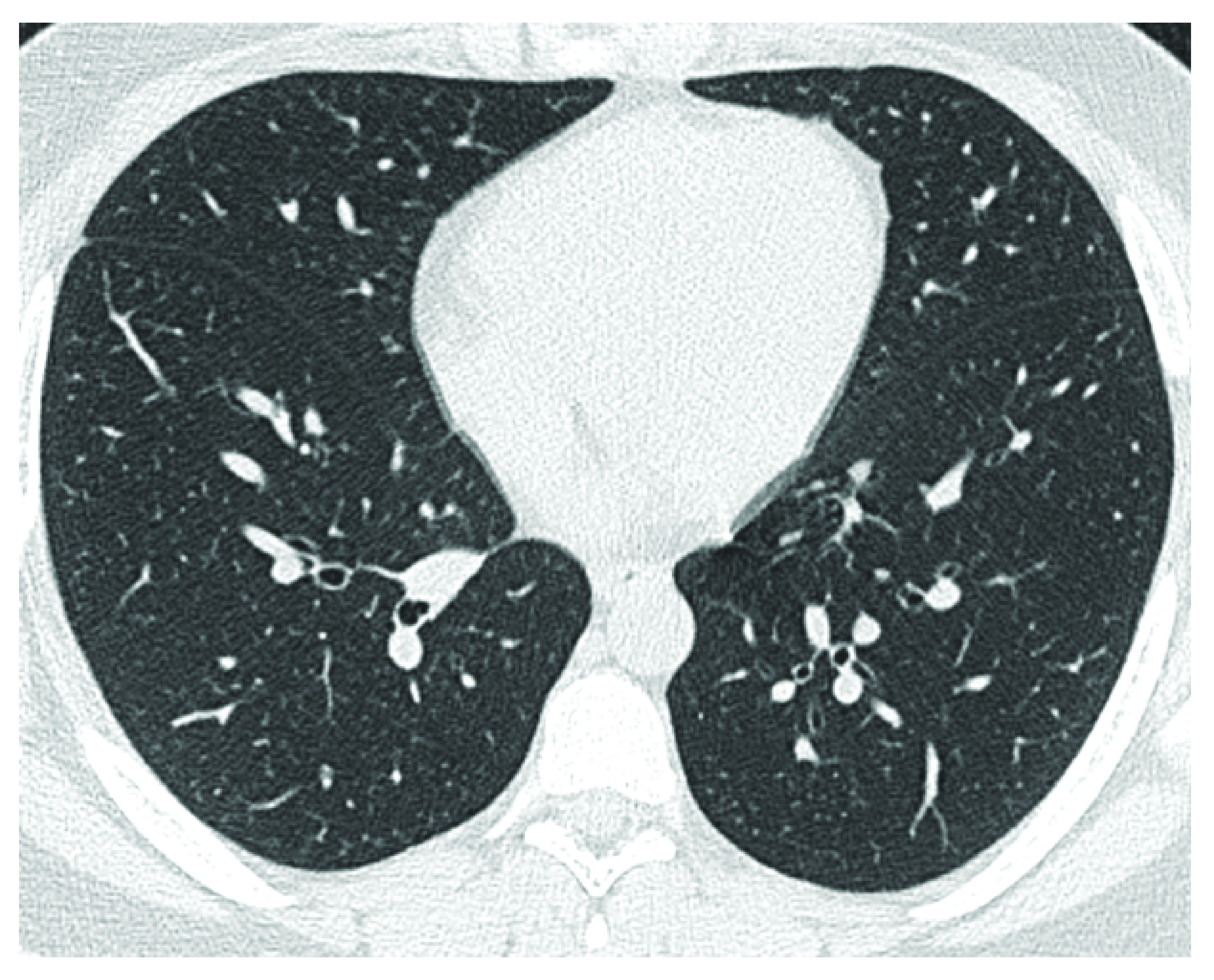
Chest computed tomography of a 16-year-old male with COVID-19 infection. Male presented with fever and cough shows normal chest computed tomography (group 1).

**Figure 2.**
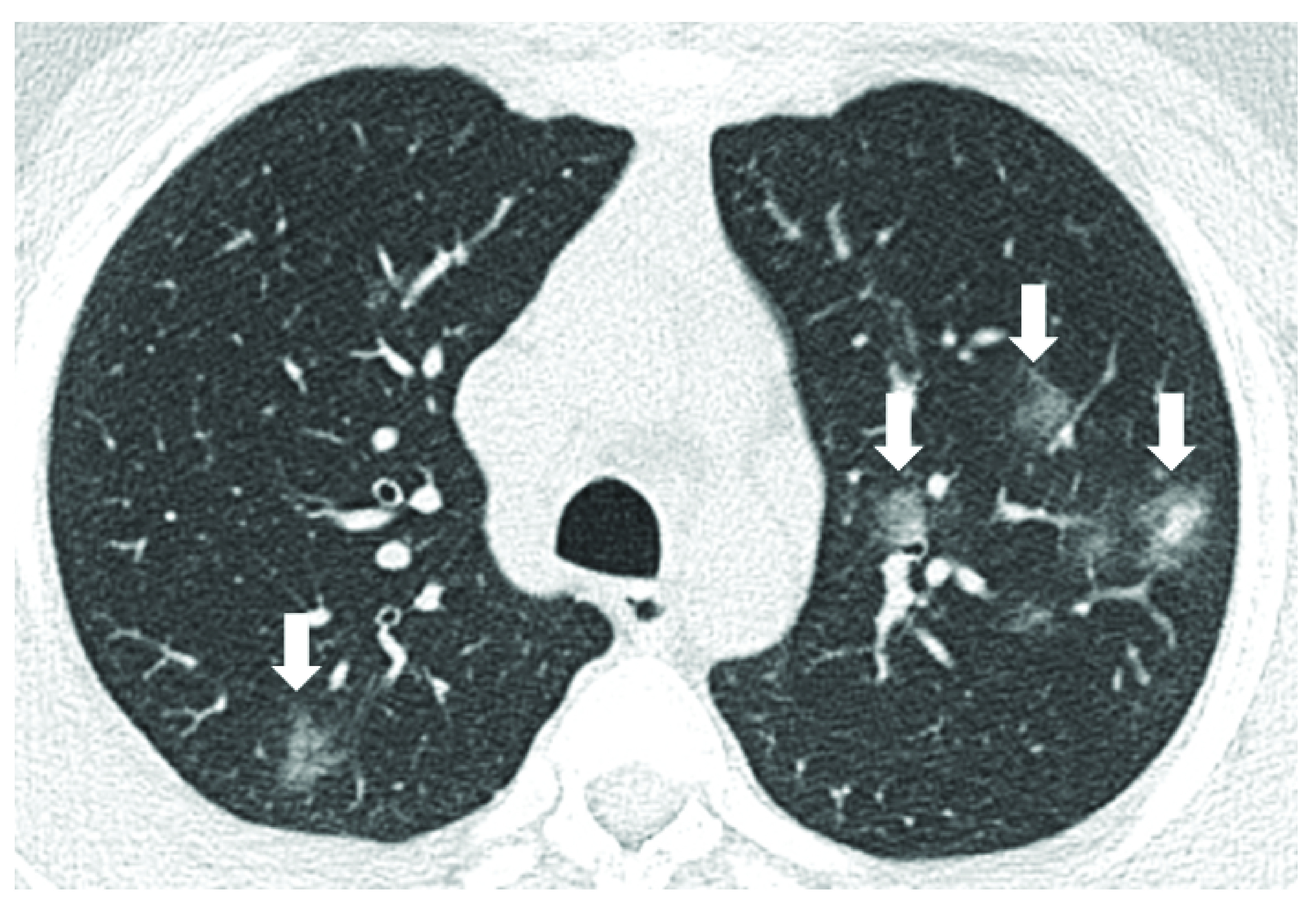
Chest computed tomography of an 18-year-old male with COVID-19 infection. Male presented with fever shows multiple bilateral asymmetrical multifocal ground-glass opacity. with consolidation in the left inferior lingular segment with evidence of halo sign (arrows) (group 2).

The two reviewers (KMD, JK) reached a consensus on all findings except for three cases where the arbitrator (TAM) was asked to make a final assessment. The chest CT was normal in 25 of 53 (47.1%) pediatric patients (Figure 1) and revealed pneumonia in 28 of 53 (52.8%) pediatric patients (Figure 2). The details of the chest CT findings are provided in Table 1. The COVID-RADS grades were as follows: Grade 1 (normal chest CT; n = 24), grade 2 (atypical findings; n = 4), grade 3 (fairly typical findings; n = 17), grade 4 (fairly typical findings and atypical findings; n = 3) and grade 5 (typical findings; n = 5). The mean dosage of radiation received was 5.6 ± 1.2 mSv (range: 2–7 mSv).

### Interobserver agreement on chest CT findings

There was almost complete interobserver agreement (IOA) (K = 0.92; p = 0.001).

### Serum biochemical parameters & its correlation with chest CT findings

**Figure 3.**
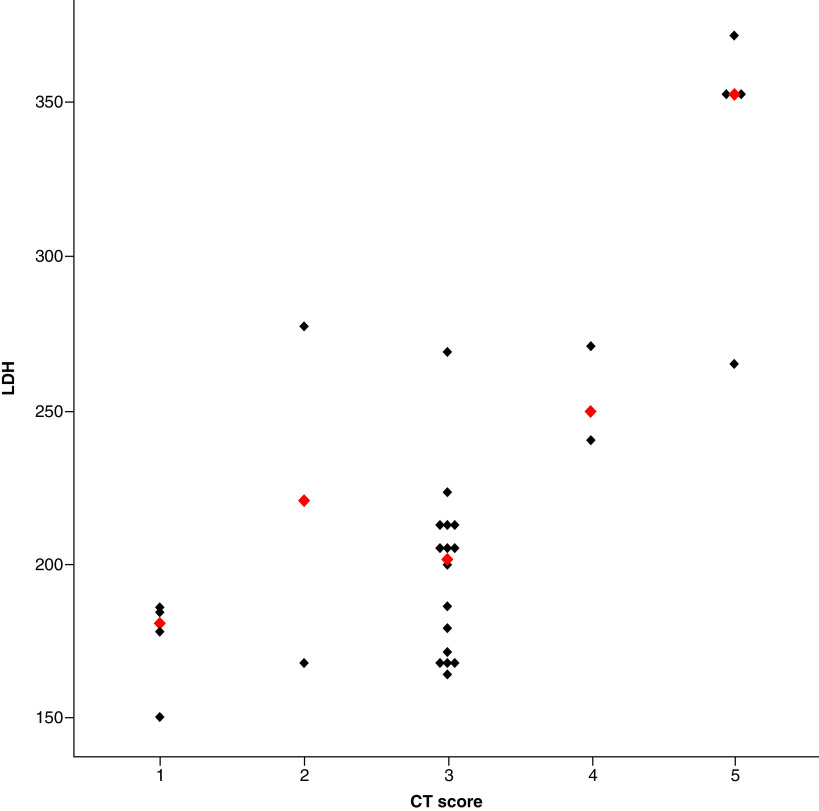
Dot plot of lactate dehydrogenase by computed tomography score in abnormal chest computed tomography score group. The red-colored diamond-shaped observations in the dot plot represent the median of lactate dehydrogenase in each computed tomography score. CT: Computed tomography; LDH: Lactate dehydrogenase.

The details of the laboratory indices are provided in Table 2. Pediatric patients in group 2 (with CT evidence for COVID-19 pneumonia) had significantly elevated levels of LDH (186.4 ± 56.5 vs 228.4 ± 60.6; p = 0.01) with a considerable increase in ferritin (62.48 ± 37.36 vs 119.56 ± 154.06, p = 0.08) and C-reactive protein (4.42 ± 8.5 vs 5.1 ± 8.98; p = 0.78) levels. Other than that, there was no statistically significant difference in patients’ white blood cells, platelets, lymphocytes, serum creatinine and ALT (Table 1). In our study, ferritin levels were skewed for normal and abnormal CT chest examinations, but there was no statistically significant difference. The value of the Eta coefficient, η = 0.706, suggests a strong correlation between LDH and COVID-RADS CT scores and 50% of LDH variance can be attributed to the respective CT score (Figure 3).

### Patient outcomes

All pediatric patients were sent to a quarantine facility for 14 days, including the hospital days. No pediatric patients exhibit any severe illnesses, and all patients had full recovery.

### Discussion

Our study establishes a clear correlation between serum biochemical parameters and CT-imaging features in children with laboratory-confirmed COVID-19 pneumonia. The group of patients with COVID-19 pneumonia had significantly elevated LDH levels (228.4 ± 60.6; p = 0.01) with a concomitant rise of ferritin (119.56 ± 154.06; p = 0.08) and C-reactive protein (5.1 ± 8.98; p = 0.78) levels. The COVID-RADS pneumonia grading system demonstrated a strong correlation with serum LDH levels (Eta coefficient test statistic = 0.706). In contrast, there were no considerable differences noted between patients’ white blood cells, platelets, lymphocytes, serum creatinine and ALT. It should be mentioned that the group of patients with a normal CT examination exhibited elevated LDH (186.4 ± 56.5) levels against normal levels in children (60–170 units/l).

According to our findings, the results of serum LDH for COVID-19 pneumonia and chest CT COVID-RADS grades for COVID-19 pneumonia were positively correlated. In addition, the serum biochemical parameters were significantly increased in group 2 of COVID-19 patients with pneumonia but were also substantially above normal levels in group 1 COVID-19 patients without pneumonia. As a result, we believe that the assessment of serum biochemical parameters is a more reliable means of detecting biochemical changes in COVID-19 patients compared with chest CT, which revealed normal findings in approximately 47% of our sample population, despite their substantially increased LDH and C-reactive protein levels.

CT scans are often nonspecific in the diagnosis of COVID-19 pneumonia [5,6,16]. During chest CT scans, children are exposed to potentially harmful ionizing radiation. Our group received radiation dosages ranging between 2 and 7 mSv. However, the associated ionization radiation is a major drawback of the CT method. Although the risk of diagnostic radiation exposure is low, it is believed to accumulate over time. This is consistent with the findings of Pearce *et al.* in a study that addressed the critical issue of whether CT scans in childhood and adolescence increase the risk of cancer in patients [17].

As a result, a substitute hematologic parameter capable of providing comparative analytic data without exposing patients to ionizing radiation would be ideal for the evaluation of pediatric COVID-19 patients. Therefore, our findings suggest that the serum biochemical parameters, especially LDH levels, may serve as a surrogate for chest CT in children with COVID-19 infection.

Since the beginning of this pandemic, CT imaging and hematologic findings in COVID-19 patients are commonly reported separately but seldom together. For example, several imaging studies on children have reported findings that do not mention serum biochemical variables [3,4,6]. Furthermore, Qiu *et al.* recently published a study where the serum biochemical parameters were measured but were not compared with the related imaging results of pediatric patients [10]. Interestingly, our study showed a substantial increase in serum LDH levels in 49% of patients with a good correlation to the COVID-RADS score. Although it is reasonable to expect that inflammatory serum biochemical parameter markers, such as serum LDH, C-receptive protein and ferritin, would be elevated in patients with any concurrent or asymptomatic infection, including COVID-19 infection, these inflammatory biomarkers are now considered important prognostic indicators in COVID-19 pneumonia [12]. Increased inflammatory markers have been linked to an increased risk of ARDS, ICU admission and mortality in patients with COVID-19 pneumonia [11,12,18]. Notably, a recent report on 36 pediatric COVID-19 patients found that serum biochemical parameters, such as lymphopenia, were significantly altered; however, an explicit assessment of LDH levels was not performed [10]. Therefore, it is not feasible to compare these findings with our results because the authors did not correlate hematologic findings with abnormal chest CT findings, which was the focus of our current study.

In China, chest CT is widely used as a COVID-19 screening tool and despite its high sensitivity, several studies have questioned its effectiveness [19,20]. A recent study comparing the added diagnostic benefit of chest CT versus chest radiography in pediatric COVID-19 pneumonia patients supports the notion that CT is not clinically recommended for the initial evaluation of mild to moderately symptomatic pediatric patients with COVID-19 pneumonia [5]. According to the American College of Radiology, the Fleischner Society and an international expert consensus statement on pediatric COVID-19 patients, imaging should be avoided in pediatric patients who test positive for COVID-19 by RT-PCR but are either asymptomatic or have mild symptoms. Chest CT should only be used in individuals with progressive disease status [6,19]. Our findings suggest that ionizing radiation imaging techniques, such as chest CT, may be unnecessary for the initial evaluation of COVID-19 children in the absence of abnormal serum biochemical parameters. Alternatively, lung ultrasound and MRI are also gaining popularity as powerful noninvasive imaging modalities that can be used in pediatric patients [21,22]. For instance, a bilateral patchy distribution of multiform clusters, interspersed with spared areas in ultrasound identified COVID-19 infection in a favorable epidemiological context [21].

Notably, 53 (94.6%) of the 56 chest CT images included in our study were also used in a previously published study, which solely focused on the correlation of chest radiographic and chest CT for the detection of abnormal thoracic findings in pediatric patients with COVID-19 pneumonia [5]. In contrast, this study primarily focuses on the correlation between serum biochemical parameters and chest CT for the identification and development of a surrogate for imaging tests.

## Limitations

Our findings are promising and noteworthy and can thus make a prominent contribution to the clinical evaluation of COVID-19 but also aid in the differentiation and prioritization of vulnerable patients for optimal management. However, due to the retrospective nature of our research, we could not schedule different investigations and; therefore, could not include and track changes of all pertinent serum biochemical parameters (procalcitonin, d-dimer and IL-6) in our study. Second, CT remains the gold standard for the detection of lung lesions and it was used in this group with radiation exposure to pediatric patients.

## Conclusion

Our study revealed a novel and significant correlation between radiological abnormalities on initial chest CT examinations and serum biochemical parameter abnormalities, specifically elevated serum LDH levels. As a result, serum biochemical markers may be used in the initial evaluation of children with suspected COVID-19 pneumonia compared with an ionizing radiation modality such as chest CT. However, and provided that patients exhibit symptoms of progressing illness, they should be examined with chest CT or with an alternative test such as ultrasound or MRI.

**Summary points**

- This study aimed to investigate whether serum biochemical parameters can be used as a surrogate for chest computed tomography (CT) in radiation-vulnerable juvenile COVID-19 patients.
- Patient radiation doses ranged from 2 to 7 mSv in each examination.
- The patients with CT-based evidence of COVID-19 pneumonia had significantly elevated lactate dehydrogenase (186.4 ± 56.5 vs 228.4 ± 60.6, p = 0.01) levels with a concomitant rise in other serum biochemical parameters.
- The value of the Eta coefficient test statistic, η = 0.706, suggests that there is a strong correlation between lactate dehydrogenase and the COVID-RADS CT score.

## References

[1] Koh HK, Geller AC, VanderWeele TJ. Deaths from COVID-19. JAMA 325(2), 133–134 (2021). 33331884 10.1001/jama.2020.25381

[2] de Souza TH, Nadal JA, Nogueira RJN, Pereira RM, Brandão MB. Clinical manifestations of children with COVID-19: a systematic review. Pediatr. Pulmonol. 55(8), 1892–1899 (2020). 32492251 10.1002/ppul.24885 PMC7300659

[3] Foust AM, McAdam AJ, Chu WC Practical guide for pediatric pulmonologists on imaging management of pediatric patients with COVID-19. Pediatr. Pulmonol. 55(9), 2213–2224 (2020). 32462724 10.1002/ppul.24870 PMC7283678

[4] Foust AM, Winant AJ, Chu WC, Das KM, Phillips GS, Lee EY. Pediatric SARS, H1N1, MERS, EVALI, and now coronavirus disease (COVID-19) pneumonia: what radiologists need to know. Am. J. Roentgenol. 215, 1–9 (2020). 32352308 10.2214/AJR.20.23267

[5] Das KM, Alkoteesh JA, Al Kaabi J Comparison of chest radiography and chest CT for evaluation of pediatric COVID-19 pneumonia: does CT add diagnostic value? Pediatr. Pulmonol. 56(6), 1409–1418 (2021). 33631061 10.1002/ppul.25313 PMC8014659

[6] Foust AM, Phillips GS, Chu WC International expert consensus statement on chest imaging in pediatric COVID-19 patient management: imaging findings, imaging study reporting and imaging study recommendations. Radiol. Cardiothorac. Imaging 2(2), e200214 (2020). 33778577 10.1148/ryct.2020200214 PMC7233446

[7] Sodhi KS, Lee EY. What all physicians should know about the potential radiation risk that computed tomography poses for paediatric patients. Acta Paediatr. 103(8), 807–811 (2014). 24673144 10.1111/apa.12644

[8] Sodhi KS, Krishna S, Saxena AK, Sinha A, Khandelwal N, Lee EY. Clinical application of ‘justification’ and ‘optimization’ principle of ALARA in pediatric CT imaging: “How many children can be protected from unnecessary radiation?”. Eur. J. Radiol. 84(9), 1752–1757 (2015). 26072096 10.1016/j.ejrad.2015.05.030

[9] MacDougall RD, Strauss KJ, Lee EY. Managing radiation dose from thoracic multidetector computed tomography in pediatric patients: background, current issues, and recommendations. Radiol. Clin. North Am. 51(4), 743–760 (2013). 23830796 10.1016/j.rcl.2013.04.007

[10] Qiu H, Wu J, Hong L, Luo Y, Song Q, Chen D. Clinical and epidemiological features of 36 children with coronavirus disease 2019 (COVID-19) in Zhejiang, China: an observational cohort study. Lancet Infect. Dis. 20(6), 689–696 (2020). 32220650 10.1016/S1473-3099(20)30198-5 PMC7158906

[11] Terpos E, Ntanasis-Stathopoulos I, Elalamy I Hematological findings and complications of COVID-19. Am. J. Hematol. 95(7), 834–847 (2020). 32282949 10.1002/ajh.25829 PMC7262337

[12] Fan BE, Chong VCL, Chan SSW Hematologic parameters in patients with COVID-19 infection. Am. J. Hematol. 95(6), E131–E134 (2020). 32129508 10.1002/ajh.25774

[13] Hansell DM, Bankier AA, MacMahon H, McLoud TC, Muller NL, Remy J. Fleischner Society: glossary of terms for thoracic imaging. Radiology 246(3), 697–722 (2008). 18195376 10.1148/radiol.2462070712

[14] Bernheim A, Mei X, Huang M Chest CT findings in coronavirus disease-19 (COVID-19): relationship to duration of infection. Radiology 295, 685–691 (2020). 10.1148/radiol.2020200463 PMC7233369 32077789

[15] Salehi S, Abedi A, Balakrishnan S, Gholamrezanezhad A. Coronavirus disease 2019 (COVID-19) imaging reporting and data system (COVID-RADS) and common lexicon: a proposal based on the imaging data of 37 studies. Eur. Radiol. 30(9), 4930–4942 (2020). 32346790 10.1007/s00330-020-06863-0 PMC7186323

[16] Rousan LA, Elobeid E, Karrar M, Khader Y. Chest x-ray findings and temporal lung changes in patients with COVID-19 pneumonia. BMC Pulm. Med. 20(1), 1–9 (2020). 32933519 10.1186/s12890-020-01286-5 PMC7491017

[17] Pearce MS, Salotti JA, Little MP Radiation exposure from CT scans in childhood and subsequent risk of leukaemia and brain tumours: a retrospective cohort study. Lancet 380(9840), 499–505 (2012). 22681860 10.1016/S0140-6736(12)60815-0 PMC3418594

[18] Zhou F, Yu T, Du R Clinical course and risk factors for mortality of adult inpatients with COVID-19 in Wuhan, China: a retrospective cohort study. Lancet 395, 1054–1062 (2020). 32171076 10.1016/S0140-6736(20)30566-3 PMC7270627

[19] Rubin GD, Ryerson CJ, Haramati LB The role of chest imaging in patient management during the COVID-19 pandemic: a multinational consensus statement from the Fleischner Society. Chest 158(1), 106–116 (2020). 32275978 10.1016/j.chest.2020.04.003 PMC7138384

[20] Chang T-H, Wu J-L, Chang L-Y. Clinical characteristics and diagnostic challenges of pediatric COVID-19: a systematic review and meta-analysis. J. Formos. Med. Assoc. 119(5), 982–989 (2020). 32307322 10.1016/j.jfma.2020.04.007 PMC7161491

[21] Poggiali E, Dacrema A, Bastoni D Can lung US help critical care clinicians in the early diagnosis of novel coronavirus (COVID-19) pneumonia? Radiology 295(3), E6–E6 (2020). 32167853 10.1148/radiol.2020200847 PMC7233381

[22] Kapur S, Bhalla AS, Jana M. Pediatric chest MRI: a review. Indian J. Pediatr. 86(9), 842–853 (2019). 30719641 10.1007/s12098-018-02852-w

